# A Treatise on Lightning Conductors; Compiled from a Work on Thunderstorms

**Published:** 1853-07

**Authors:** 


ART. VII.—*A Treatise on Lightning Conductors;* compiled from a work on Thunderstorms, by S. W. HARRIS, F. R. S., and other standard authors. By LUCIUS LYON, A. M. New York: George P. Putnam. 1853.

We need scarcely make an apology for noticing this work in the Review department of the Western Journal of Medicine and Surgery. Medical men are often consulted on the various subjects connected with the use of lightning rods, because the people, who are ignorant on all the field of electrical matters, think that medical men should be familiar with everything on the subject. It is a source of regret that medical men are not always as much entitled to this confidence as they should and could be, and if they do not confess ignorance, they do worse—they give erroneous advice which, when faithfully followed may end in loss of life to the credulous inquirer. It must be confessed that grievous errors on electrical subjects prevail in many works of authority; facts are misstated, inferences are wrongly drawn, assumption takes the place of established truth, and presumption usurps the position of science. Yet, when we look at the disastrous powers of lightning, and know how often it destroys life by an instantaneous effect, we see how necessary it is that every human being who feels interested in living shall know the whole truth on the subject of electrical laws. The mind of man is capable of disarming this terrible agent of its destructive powers, and to that important point every consideration should be given.

The treatise before us is a very thorough and satisfactory exposition of all that species of knowledge that is needed upon the subjects of electricity, and the means of guarding against its evils. The author very truly and properly says:

"There can be no doubt of the utility of such a book as is here presented. After expending large sums of money for the most approved apparatus of rods, many persons have been bitterly disappointed in their expectations, and have received no adequate return for their outlays. The truth is, that our countrymen have not acted on this subject with that shrewdness for which, in other things, they are proverbial. They have taken too much upon trust, and neglected the investigation of the facts. The iron conductors, put up at no inconsiderable cost, and supposed to secure perfect safety to the structures which they surmount, instead of proving themselves faithful sentinels and guardians in the hour of peril, have too often turned traitors, and invited the destruction which they promised to avert. The "swift fire of Jove, hurled by his red right arm," has not unfrequently bolted from the iron track, prepared for its harmless descent, and, indignant at some defective joint, or sudden break, or the want of sufficient metal for its free discharge, has made a forcible passage for itself, often leaving shattered walls, and chimneys, and blazing roofs, to attest its terrific power. Happy for the sufferers if the blasted corpse of some loved one has not added unutterable woe to the desolation of the scene!

Such disastrous consequences are due, in part, to imposture, but far more frequently to the ignorance and carelessness of workmen, and to the blind credulity of those who have employed them. In many instances, too, the owners of property have suffered an apparatus, originally well adjusted, to get out of repair. The distrust which might have arisen from these accidents, has no doubt been mitigated or prevented, in a great degree, by the difficulty of detecting any defect in a system of conductors—a philosophical knowledge of the scienc of electricity often being requisite for doing so.

Partly from the imperfections alluded to above, but much more from the unnacountable apathy which exists on the whole subject, a very large proportion of the owners of buildings in this country, and probably a still greater proportion in other countries, have neglected to avail themselves of the lightning conductor in any form. An incalculable amount of property, and many lives, which might be made at least relatively secure, are thus constantly and needlessly jeopardized. How great a risk is incurred, and how nearly absolute safety is attainable by conductors, the author hopes to demonstrate in the following pages."

The work opens with a consise, or rather a condensed series of views of natural and artificial electricity, which contain some of the best arranged truths on the subject that we have met. This portion of the work deserves thorough study by all who are interested in the phenomena of electricity, and who is not? We can scarcely find any portion of this part of the work that can be abstracted from the rest, because of the intimate relations that all the parts hold towards one another. But we must ask the reader's attention to the following, on account of its great interest, and because the phenomena thus detailed are not sufficiently known and understood. But a few weeks since, about the 17th of May, there was an exhibition of the phase of electricity set forth in this extract, in a lumber yard in this city. There were four persons in the vicinity of the disaster. One of the four was driving a wagon, and he sat on one of the four horses; another was on the hounds of the wagon, another walking behind the wagon, and a fourth was near another wagon. An ascending stroke of electricity killed the negro driver, and the horses of his team, and lifted the wagon two feet up, above the ground. The man on the hounds was uninjured. The man between the two wagons was struck across the abdomen, and felt a burning sensation for some hours after, and the fourth felt a similar sensation in one of his legs.

The phenomena of the ascending stroke are thus set forth :

Sometimes the thunderbolt passes from the earth to the clouds, and in this case it is called by some philosophers the "ascending stroke," by others "the returning stroke." Facts are not wanting to indicate the progress of electricity upward. The Marquis Maffei was the first who observed this curious phenomenon. He distinctly saw, during a storm, the lightning issue from the ground with a loud noise. The Abbe Lioni and M. Seguier of Nismes, saw the lightning rise in the form of flame six feet high, followed by a loud noise.

One of the most interesting cases of the ascending stroke has been recorded by John Williams, Esq. It took place upon the hills above the village of Great Malvern, on the 1st July, 1826. A party had taken refuge from the storm in a circular building roofed with sheet iron, and one of the ladies on entering the hut expressed her alarm lest the lightning should be attracted by the iron roof. They had scarcely entered their retreat, and were about to partake of some refreshment, when a violent storm of thunder and lightning came on from the west. About forty-five minutes past two, a gentleman who stood at the eastern entrance, saw a ball of fire which seemed to him moving on the surface of the ground. It instantly entered the hut, forcing him several paces forword from the doorway. On his recovering from the shock, he found his sisters on the floor of the hut, fainting, as he imagined, from terror. Two of the ladies had died instantly; another lady, and the rest of the party, were much injured. The explosion which followed the flash of lightning, was said, by the inhabitants of the village, to have been terrific. Mr. Williams, who immediately examined the hut, found a large crack in the west side of the building, which passed upward from near the ground to the frame of a small window above which the iron roof was a little indented. Mr. Williams conceived it to be quite clear, from the place of the fragments of stone and other appearances, that the clouds were negatively electrified during the storm.*

A very curious instance of the ascending stroke is related by G. F. Richter, in his work on thunder. He informs us that in the cellar belonging to the Benedictine monks of Fontigno, while the servants were employed in pouring into a cask some wine which had been just boiled, a fine light flame appeared round the funnel, and they had scarcely finished their operations, when a noise like thunder was heard; the cellar was instantly filled with fire; the cask was burst open, although hooped with iron, the staves were thrown with prodigious violence

* *Encyclopædia Britannia*, vol. 8, p. 619.

against the wall, and, on examination, a hole of three inches diameter was found in the bottom of the cask.*

On the 24th of February, 1774, lightning struck the steeple of the village of Rouvroi, to the northwest of Arras. A pavement composed of large blue stones, which was laid under the steeple was violently raised upward.

In the summer of 1787 lightning struck two persons who had taken refuge under a tree near the village of Tacon, in Beaujalois. Their hair was *driven upward and found upon the top of the tree.* A ring of iron which was upon the shoe of one of these persons was found afterwards *suspended on one of the upper branches.*

On the 29th of August, 1808, lightning struck a small building near the hospital of Salpétrière in Paris. A laborer who was in it was killed, and after the event the pieces of his hat were found incrusted on the ceiling of the room.

When trees have been barked by lightning it frequently happens that the bark is stripped from the base *upward* to a certain height, and the upper part of the tree is untouched. This occurred with several trees in the Champs Elysées, at Paris, in a storm which took place in June, 1778.

The leaves of trees which have been struck by lightning often exhibit the effects of heat on their lower surfaces, but not at all on their superior surfaces.

Among the numerous manifestations of the discharge of electric matter from the surface of the earth, one of the most circumstantial and authentic is due to Brydone, who, being on the spot where the occurences took place, was in part witness to them, and collected the particulars from other eye-witnesses with scrupulous care.

On the 10th July, 1785, a storm broke out between noon and one o'clock, in the neighborhood of Coldstream. During its continuance, there occurred in the surrounding country several remarkable accidents.

A woman who was cutting grass on the banks of the *Tweed*, was suddenly thrown down without any apparent cause. She called her companions immediately to her aid, and told them that she received a sudden and violent blow on the soles of her feet, but whence it proceeded

* *London Encyclopædia*, vol. 8, p. 59.

she could not tell. At the moment this happened there was neither thunder nor lightning.

A shepherd attached to a farm called Lennel Hill, saw a sheep suddenly fall, which the moment before appeared in perfect health. He ran to raise it from the ground, and found it stiff dead. The storm was then approaching, but distant.

Two coal wagons, driven by two boys, seated on the benches in front of them, had just crossed the *Tweed*, and were in the act of ascending a hill on the banks of the river, when a loud explosion was heard like the report of several guns fired nearly together, and unattended by any rolling or continued sound like that which usually accompanies thunder. At the moment of this explosion the boy who drove the second wagon saw the foremost wagon with the two horses and driver suddenly fall to the ground, the coal being scattered about in all directions. On examination, the driver and horses were found to be stiff dead. The coal which was dispersed had the appearance of having been for some time in the fire. At the points where the tires of the wheels rested at the time of the explosion, the ground was found to be pierced by two circular holes which being examined by *Brydone* half an hour after the occurrence, emitted a strong odor resembling that of ether. The tires of the wheels showed evident marks of fusion at the points which were in contact with the road at the moment of the explosion, and at no other part. The hair was singed on the legs and under the bellies of the horses, and by a careful examination of the marks left in the dust of the road where they fell, it was apparent that they must have been struck suddenly stone dead, so that no life remained when they touched the ground. Had there been any convulsive struggle, the marks would have been visible. The body of the driver was scorched in different places, and his dress, shirt, and particularly his hat, were reduced to rags. A strong odor proceeded from them.

All the witnesses of this occurrence agreed, that no luminous appearance whatever attended it. The driver of the second wagon was conversing with his comrade, and was looking toward him at the moment he was struck down, being at about twenty yards behind him, but saw no light. A shepherd standing in an adjacent field, told

Mr. Brydone that he had his eye on the wagon at the very instant of the explosion, but he saw no light. He saw a vortex of dust arise at the place of the explosion, but unaccompanied by any luminous appearance. Finally, Mr. Brydone himself at the moment of the event was standing at an open window, with a watch in his hand, explaining to the persons around him the method of calculating the distance of the lightning by observing the interval between the flash and the thunder, and he heard the explosion, but perceived no light.*

Professor Dewey, D. D., of Rochester University, related to the writer some few months since, that during his connection with Williamstown College, Mass., a church in South Williamstown was struck by lightning. The house was carefully examined by himself after it was struck, when he saw leaves of flowers that had grown by the walls of the church, lodged in the cobwebs up under the eaves.

Some time last summer Mr. Platt's house, Deep River, Connecticut, was struck by lightning. After the accident, the house was carefully examined by myself and many others. It was evidently an instance of the "ascending stroke." The front part of the house, including the entire length of the wing attached was traversed by the lightning, leaving marks of terrific violence wholly unaccountable, unless done by an upward force.

We have multiplied the number of instances under this head, for two reasons: First, because so many of those little read in electrical science utterly disbelieve the existence of an upward stroke, and even laugh at the idea as absurd and whimsical. Secondly, because an intelligent belief in an upward stroke has an important relation to the application of lightning conductors for the protection of buildings, as we shall presently see.

Various electrical phenomena, of a very interesting kind, have been observed by travellers, when ascending lofty mountains. In 1767, M. M. Saussure, Pictet, and Jallabert, when on the top of Mount Breven, received small electric shocks at their finger-ends, by stretching out their arms, and a whistling noise even accompanied them. The gold button on M. Saussure's hat yielded

---

* Dr. Lardner's *Lectures on Science and Art*, vol. II., p. 72.

distinct sparks. In 1814, a party of Englishmen experienced similar effects on Mount Ætna, during a storm of thunder and lightning, accompanied by a fall of snow. One of the party felt his hair moving, and upon raising his hand to his head, a buzzing sound issued from his fingers. The rest of the party experienced the same sensations, and by moving their hands and fingers they produced a variety of musical sounds, audible at the distance of forty feet. On the 27th of June, 1825, Dr. Hooker and a party of botanists witnessed effects like those descr.bed, during a fall of snow on Ben-Nevis, when there was no thunderstorm The snow fell very heavily for nearly two hours. Soon after it began, a hissing sound was heard every where around them, and continued about an hour and a half. It seemed to proceed from every point in the vicinity; and on arriving at the cairn on the summit of the mountain, they could almost determine the stones from which the electricity issued. The hair of several of the party exhibited, when touched, the usual electrical phenomena.*

The second section of the work is devoted to the "Application of metallic conductors to the defence of buildings and shipping from lightning," and every page is full of interest. We make room for the observations on the "Nature of lightning rods:"

The damage sustained by buildings and ships in thunderstorms being invariably found to occur in the spaces where good conductors of electricity cease to be continued, it became an important question in practical science, how far it would be desirable to provide at once a continuous line of conduction, through which the electrical discharge might be transmitted without any immediate explosion, and consequently without damage to the general mass. An idea first suggested by the American philosopher, Franklin, and since carried effectually into practice under the form of a lightning rod.

The application of such a rod to a building or ship, is evidently equivalent to the uniting into a continuous conducting train, all the detached metallic masses between

---

* *Encyclopædia Britannica,* vol. viii., p, 620, which damage ensues, and in the case of particular buildings, into the construction of which such masses do not enter, it supplies the degree of conducting power requisite for their safety. A conducting rod, therefore, in whatever way it may be applied, is to be considered merely as a means of perfecting the conducting power of the whole mass, so as to admit of intense discharges of lightning being securely transmitted, which otherwise would not pass without intermediate explosion and damage; for it must never be forgotten, as an important feature in the consideration of this question, that the materials of which buildings and ships are composed, are for the most part such as come under the denomination of conductors (p. 8); the whole fabric is, therefore, to a greater or less extent, an electrical conductor. Now the chance of its escaping damage from a discharge of lightning, increases with its power of transmitting the electrical action by which it is assailed. If we could suppose a ship or building to have a perfect conducting power in all its parts, or if we imagine it to be metallic throughout, then damage from lightning would be unknown. Thus discharges of lightning struck repeatedly on the iron steamboat which accompanied Lander in his last attempt to explore the interior of Africa, without producing the slightest effect on it; whilst vessels built of wood and metal were damaged. A man in armor would certainly be safe in a thunderstorm, from the great conducting power of the metal as compared with the human body. The great object, therefore, to be attained in the application of lightning conductors to the defence of buildings and shipping from lightning, is to bring the general mass as nearly into this state as possible.

It has already been observed (p. 9), that Mr. Cavendish determined the conducting power of iron to be, in relation to that of water, as four hundred millions to one. Taking therefore the conducting power of water as unity, and assuming it as the value of the mean conducting power of the general mass of a building, which is upon the whole not far from the truth, then, by giving a building a lightning conductor of great capacity, and connecting it with the detached masses of metal as far as is possible, we multiply its chance of escaping damage, in the intermediate points in which the conductor is applied, four hundred millions of times; and considering that by the laws of electrical action, the electrical discharge finds its way through any line or lines, which upon the whole offer the least resistance to its passage, nothing can be clearer than the deduction, that any incapacity of the building as a whole, to transmit the charge through its parts without explosion, would be supplied by the presence of the conductor; and hence this multiplied chance of escape applies equally to the whole building. It is therefore demonstrable by physical facts, that perfect security is to be derived from an efficient conductor properly applied.

The first lightning conductor seen in England was erected by Dr. Watson at Payneshill, in 1762. In 1769 the Jacob tower at Hamburg was defended by a lightning rod, and after the cathedral church at Sienna had been repeatedly damaged by lightning, a similar means of protection was employed there also. The inhabitants first regarded the rod with terror and apprehension, and called it a Heretic Rod; but on the 10th of April, 1777, a thunderstorm occurred, and there fell on the tower a heavy stroke of lightning, which was carried off by the rod, without doing the slightest damage even to the gilded ornaments near which it passed.* The inhabitants now began to look on the heretic rod with more confidence; and it is an important fact, that this church does not seem to have suffered from lightning since.

But our waning space admonishes us to rest the subject here. We shall resume and finish the review in our next number.

---

* Marbuch, *Enkyklopædia*, vol. i., p 314.

